# The Impact of High Levels of Compensatory Exercise on Treatment Outcomes in Threshold and Subthreshold Bulimia Nervosa

**DOI:** 10.3390/nu16142337

**Published:** 2024-07-19

**Authors:** Lucía Camacho-Barcia, Isabel Sánchez, Ana Ibáñez-Caparrós, Noriaki Ohsako, Roser Granero, Cristina Artero, José Manuel Crespo, Georgios Paslakis, Susana Jiménez-Murcia, Fernando Fernández-Aranda

**Affiliations:** 1 Clinical Psychology Department, Bellvitge University Hospital, 08907 Barcelona, Spain; lcamacho@idibell.cat (L.C.-B.); isasanchez@bellvitgehospital.cat (I.S.); cartero@idibell.cat (C.A.); sjimenez@bellvitgehospital.cat (S.J.-M.); 2 Psychoneurobiology of Eating and Addictive Behaviours Group, Neurosciences Programme, Bellvitge Biomedical Research Institute (IDIBELL), 08908 Barcelona, Spain; nohsako@idibell.cat (N.O.); roser.granero@uab.cat (R.G.); 3 Ciber Fisiopatología Obesidad y Nutrición (CIBERobn), Instituto de Salud Carlos III, 08908 Barcelona, Spain; 4 Department of Psychiatry, University Hospital Germans Trias i Pujol, 08916 Badalona, Spain; aibanezc.germanstrias@gencat.cat; 5 Institut Recerca Germans Trias i Pujol (IGTP), 08916 Badalona, Spain; 6 Department of Psychiatrics and Legal Medicine, School of Medicine, Autonomous University of Barcelona, 08193 Barcelona, Spain; 7 Department of Psychiatry, Graduate School of Medicine, Chiba University, Chiba 263-8522, Japan; 8 Departament de Psicobiologia i Metodologia de les Ciències de la Salut, Universitat Autònoma de Barcelona, 08193 Barcelona, Spain; 9 Biomedical Research Networking Centre in Mental Health (CIBERSAM), 28029 Madrid, Spain; jmcrespo@bellvitgehospital.cat; 10 Departament of Psychiatry, Bellvitge University Hospital, Bellvitge Biomedical Research Institute-Idibell, 08907 Barcelona, Spain; 11 Department of Clinical Sciences, School of Medicine and Health Sciences, University of Barcelona, Bellvitge Campus, L’Hospitalet de Llobregat, 08907 Barcelona, Spain; 12 Psychiatry and Mental Health Group, Neurosciences Programme, Bellvitge Biomedical Research Institute (IDIBELL), 08908 Barcelona, Spain; 13 University Clinic for Psychosomatic Medicine and Psychotherapy, Ruhr-University Bochum, Medical Faculty, Campus East-Westphalia, 32312 Luebbecke, Germany; georgios.paslakis@ruhr-uni-bochum.de; 14 Centre for Psychological Services, University of Barcelona (UB), 08035 Barcelona, Spain

**Keywords:** bulimia nervosa, subthreshold bulimia nervosa, compensatory exercise, eating disorders, treatment outcome

## Abstract

Bulimia nervosa (BN) and other specific feeding or eating disorders with subthreshold BN symptoms (OSFED-BN) are characterized by recurrent binge eating episodes accompanied by compensatory behaviors, including excessive exercise. We aimed to examine the role of compensatory exercise on several clinical disorder-related variables and the treatment outcomes. The sample included 478 patients diagnosed with either BN or OSFED-BN admitted for a 16-week eating disorder-specific treatment program. A battery of questionnaires was administered to evaluate eating and general psychopathology, and personality traits. Other clinical disorder-related data, including levels of compensatory exercise, were assessed through a semi-structured clinical interview. Between-group comparisons of compensatory exercise levels were analyzed, as a predictive model of risk of poor treatment outcomes. Path analysis was conducted using structural equation models to estimate the direct and indirect effects between the main variables. Higher levels of self-reported compensatory exercise were associated with greater eating psychopathology, general psychopathology, and more dysfunctional personality traits and were a predictor of poor treatment outcomes. Additionally, these levels achieved a mediating role in several paths contributing to a higher likelihood of a poor outcome. Further research is required to determine how psychotherapeutic approaches can be optimized to adequately include adaptive exercise for these patients.

## 1. Introduction

Bulimia nervosa (BN) and other specific feeding or eating disorders with subthreshold bulimia nervosa symptoms (OSFED-BN) are eating disorders (ED) characterized by recurrent binge eating episodes, accompanied by a feeling of loss of control and inappropriate compensatory behaviors to prevent weight gain, including self-induced vomiting, laxative abuse, and a high exercise load [1]. The lifetime prevalence of BN is estimated to be between 1 and 2% and approximately 4.4% for OSFED-BN [2]. Both conditions typically have their onset early in life; yet, diagnoses may be delayed due to secrecy and shame associated with binge and/or purge behaviors [3], which can lead to persistent deteriorations in the global functioning of these individuals. Interestingly, research findings suggest that OSFED-BN is not any less clinically severe or impairing than the full-blown BN [4] and that both have high rates of comorbid somatic [5,6,7] and psychiatric consequences [8,9,10], which impair prognoses [11]. Treatment approaches are multidimensional and include nutritional interventions, psychological therapy (cognitive–behavioral therapy, family therapy, or interpersonal therapy) [10,12,13], and pharmacological agents (i.e., fluoxetine) [14,15]. Nonetheless, dropout rates are high [16].

Maladaptive exercise has been associated with ED since its initial description [17], depicting an important factor affecting the development and maintenance of the disorders [18]. Compensatory exercise as the performance of exercise specifically to counteract the effects of binges on weight and shape [19] has been associated with ED subtypes characterized by high impulsivity, such as BN and OSFED-BN [19,20] and indirectly with a greater severity of ED psychopathology [21]. An estimated 55% of individuals with BN engages in maladaptive exercise [19]. Previous studies have shown that maladaptive exercise is associated with more severe eating psychopathology, prolonged hospitalizations and increased suicidal behavior in patients with ED [22,23]. These findings suggest that compensatory exercise may be associated with impaired short-term response to BN treatment and poor long-term outcomes. A recent study that compared individuals who engaged in compensatory exercise, compulsive exercise, compensatory/compulsive exercise, adaptative exercise, and non-exercise, found that compulsive and compensatory exercise as well as no exercise at baseline were all associated with significantly higher global eating psychopathology after treatment [24].

Although maladaptive exercise in its various forms has been previously studied in the context of ED, there is a still a lack of studies addressing compensatory exercise in BN and OSFED-BN, as well as its association with further clinical variables and its impact upon treatment outcomes. Thus, deepening our understanding of compensatory exercise in BN and OSFED-BN may prove useful to better assess the impact of compensatory exercise on the progression and outcomes of the disorder, and consequently help improve treatment strategies.

In light of the aforementioned considerations, the principal objectives of this study were as follows: (a) to examine the role of compensatory exercise, in BN and OSFED-BN, with regard to clinical variables (ED psychopathology, general psychopathology, illness duration, the number of binge eating episodes, and body mass index (BMI)) and personality traits and (b) to explore the impact of high levels of compensatory exercise on the outcomes of treatment. We hypothesized that higher compensatory exercise would be positively associated with higher eating-disorder-related psychopathology and with worse therapy response.

## 2. Materials and Methods

### 2.1. Participants and Procedures

The sample comprised a total of n = 478 patients who were consecutively admitted for treatment at the adult-specialized Eating Disorders Unit of the Bellvitge University Hospital in Barcelona, Spain, between July 2007 and September 2021. All individuals were diagnosed in accordance with DSM-5 criteria [1], based on a semi-structured interview conducted by experienced clinical psychologists and psychiatrists [25]. The final sample consisted of patients diagnosed with either BN (n = 389) or OSFED-BN (n = 89). In order to be included in this study, participants were required to meet the following criteria: (1) be at least 18 years of age; (2) have been diagnosed with either BN or OSFED-BN, (3) not have a learning or intellectual disability; (4) sign the informed consent form; and (5) complete the questionnaires for the assessment in their entirety. Individuals with a diagnosis of another ED type and those who did not meet the established inclusion criteria or did not engage with the treatment for a minimum of three consecutive sessions were excluded from this study. This study was approved by the Ethics Committee of the University Hospital of Bellvitge Committee (protocol code 2021/2613) and was conducted in accordance with the Declaration of Helsinki. All subjects were informed of the nature of this study and provided informed consent prior to participation.

### 2.2. Assessments

#### 2.2.1. ED-Related Clinical Variables and Compensatory Exercise

The data regarding clinical ED-related information were assessed through a semi-structured interview conducted by experienced clinical psychologists and psychiatrists, which included age of ED onset, ED duration, and the number of weekly binge/purge episodes.

A trained nurse conducted anthropometric assessments of all patients during the initial hospital interview, prior to the beginning of treatment. Weight was determined using a calibrated scale, with patients positioned in underwear and without shoes, in a non-fasting state. Height was measured using a stadiometer, with patients positioned without shoes. These values were later used to calculate the baseline body mass index (BMI) (kg/m^2^).

Information regarding compensatory exercise was obtained through a face-to-face interview with expert clinicians. Participants were requested to provide comprehensive details regarding their compensatory behaviors, including the number of hours per week engaging in compensatory exercise, over the previous month prior to the interview. This information was classified according to different categories of the clinical protocol: high (>1 h/day), moderate (between 6 and 4 h/week), and low (<4 h/week). These cut-off points were set in accordance with other classifications of high-level exercise in ED [26,27] and the World Health Organization (WHO) guidelines for physical activity [28].

#### 2.2.2. Psychometric Assessments

The following battery of questionnaires to evaluate ED symptomatology, personality traits, and general psychopathology was administered:

The Eating Disorders Inventory-2 (EDI-2) [29] is a 91-item self-report questionnaire that is used habitually to assess cognitive and behavioral characteristics of ED. The instrument measures 11 subscales including the following: drive for thinness, body dissatisfaction, bulimia, ineffectiveness, perfectionism, interpersonal distrust, interoceptive awareness, maturity fears, asceticism, impulse regulation, and social insecurity. The EDI-2 total score measures global ED severity. A Spanish population validation [30] has shown a mean internal consistency of 0.63 (Cronbach’s alpha). The internal consistency in the present sample (Cronbach’s alpha) ranged from adequate (=0.709 for “ascetic”) to excellent (=0.949 for the “total score”).

The Temperament and Character Inventory-Revised (TCI-R) [31] is a 240-item questionnaire based on the Cloninger model of personality. Four temperaments—harm avoidance, novelty seeking, reward dependence, and persistence—as well as three character dimensions of personality—self-directedness, cooperativeness, and self-transcendence—are measured. A Spanish validation in the adult population is available [32]. The internal consistency in the present sample (Cronbach’s alpha) was between adequate (=0.807 for “novelty seeking”) to excellent (=0.902 for “harm avoidance”).

The Symptom Checklist-90-Revised (SCL-90) [33] is a self-report 90-item questionnaire used to explore general psychopathology. Nine primary symptom dimensions are measured, including the following: somatization, obsession–compulsion, interpersonal sensitivity, depression, anxiety, hostility, phobic anxiety, paranoid ideation, and psychoticism. In addition, three global indices are available: the global severity index (GSI) evaluating overall distress; the positive symptom distress index (PSDI), which indicates the intensity of the symptoms; and the positive symptom total (PST), which assesses self-reported symptoms. The mean internal consistency (Cronbach’s alpha) obtained for the Spanish population [34] was 0.75. The internal consistency in the present sample (Cronbach’s alpha) ranged from adequate (=0.749 for “paranoia”) to excellent (=0.976 for the global indexes).

#### 2.2.3. Treatment and Outcome Measurements

The treatment for patients with BN and OSFED-BN consisted of 16 weekly sessions of manualized outpatient group therapy. Each session had a duration of 90 min and was led by experienced clinical psychologists. The goals of the treatment were to train patients in problem-solving strategies, cognitive restructuring, emotion regulation, improving self-esteem and body image, and relapse prevention strategies. Furthermore, the treatment also addressed the monitoring of eating behaviors, the establishment of regular nutritional patterns, and the facilitation of knowledge about the negative consequences of ED. This program has been published in Spanish [12] and has been applied with proven efficacy [35,36].

At the final stage of the treatment, patients were subject to a clinical reassessment that led to their categorization into three categories, according to the DSM-5 criteria [1]: “remission”, “partial remission”, and “non-remission”. Remission was defined as the complete absence of symptoms for a minimum of four consecutive weeks. Partial remission was defined as a significant symptomatic improvement despite the persistence of residual ED symptoms. Patients who continued to report significant symptoms and displayed dysfunctional ED-related behaviors were classified as non-remission. Patients who discontinued treatment on their own initiative were counted as dropouts. Finally, patients who achieved complete or partial remission were considered to have achieved a “good” outcome. In contrast, patients who were either non-remitters or dropouts were classified as having a “poor” outcome.

### 2.3. Statistical Analysis

Stata18 for Windows was used for the statistical analyses. The comparison of the baseline state between the groups defined by the compensatory exercise activity level was performed with an analysis of variance (ANOVA), and standardized Cohen’s *d* coefficients estimated the effect size for the pairwise comparisons (|*d*| > 0.50 was considered a mild–moderate effect and |*d*| > 0.80 a high–large effect). Chi-square (χ^2^) compared the therapy outcomes, and Cramer’s *V* coefficient measured the effect size of the proportion differences (*V* > 0.20 was considered mild–moderate effect, and *V* > 0.60 was considered a high–large effect). For these comparisons, the Finner method procedure was employed for controlling the familywise error rate [37], defining a significance level of α = 0.05.

A predictive model for the risk of poor outcomes was obtained through stepwise logistic regression, considering a number of potential predictors: participants’ sex and age, duration of the ED, ED symptom severity (EDI-2 total), body dissatisfaction levels, drive for thinness levels, number of binge episodes, BMI, compensatory exercise levels, and personality profiles (as measured by the TCI-R). The Hosmer–Lemeshow test valued the goodness of fit for the model, and the Nagelkerke’s pseudo R^2^ estimated the global predictive capacity.

Path analysis estimated the direct and indirect effects (including mediational links) between the main variables of this study. This analysis was implemented through structural equation models (SEM), defining the maximum likelihood as the estimator. To obtain the most parsimonious model with the easiest interpretation capacity, a first model including all parameters was defined, and the non-significant coefficients were deleted next (with the limitation to achieve adequate fitting). Adequate goodness of fit was considered for a root-mean-squared error RMSEA < 0.08, comparative fit index CFI > 0.90, Tucker–Lewis index TLI > 0.90, and standardized root-mean-squared residual SRMR < 0.10. The global predictive capacity of the final model was estimated with the coefficient of determination (CD).

The calculation of the global statistical power associated to the ANOVA procedures has been carried out for this study based on the comparison of 3 independent groups, which allows defining a total of 3 multiple comparisons (pairwise comparisons) and the following criteria: total sample size n = 478, maximum error variance of σ = 190 (value based on analyses of T-standardized measures, common in clinical settings), differences around δ = 5 (mean values are expected to increase from subclinical T = 60 to T = 70 range, depending on the exposure level), and significance level of α = 5% [command in Stata: *power oneway* 70 65 60, *varerror* (190) contrast (1 −1 0) n (478)]. The sample size was also adequate for the predictive analysis and the SEM, based on the studies supporting that data shape is clearly identified and accurate inference is stable (even with high variance) at N ≥ 25 observations per variable or parameter estimation [38,39].

## 3. Results

### 3.1. Description of the Sample

The majority of the participants in this study were women (92.1%), with a mean age of 29.5 years (SD = 9.6). The mean age of onset of ED-related symptoms was 19.5 years (SD = 7.9), and the mean duration of illness was 10.1 years (SD = 8.2). The number of participants who reported a high compensatory exercise level was n = 270 (56.5%), that for the participants with mild–moderate compensatory exercise was n = 84 (17.6%), and that for the participants with low compensatory exercise was n = 124 (25.9%). Regarding the treatment outcomes, the risk of good response was around 40.2%. Table 1 presents the complete description of the study variables for the complete sample: with the frequency distribution (count and percentages) for categorical variables and the mean and standard deviation (SD) for quantitative measures.

### 3.2. Baseline Comparisons between Groups of Compensatory Exercise Levels

The results of the ANOVAs comparing clinical profiles at baseline between the groups (defined according to their compensatory exercise levels) are displayed in Table 2. The first part of the table includes the mean and the SD within each group, and the second part includes the pairwise comparisons (contrasts). Statistically significant and effect size within the mild–moderate to high–large range are outlined in bold. Overall, the highest compensatory exercise levels were associated with the oldest age, longest duration of the ED-related problems, highest BMI, and highest mean for the number of binges. With regard to the severity of the ED, the highest compensatory activity levels were associated with the highest mean values in several of the EDI-2 scales, specifically for bulimia, interpersonal distrust, ineffectiveness, social insecurity, and total score. In contrast, the patients in the group with mild–moderate compensatory exercise levels reported the lowest mean scores for the EDI-2 subscales of interoceptive awareness, impulse regulation, and asceticism. The most unfavorable psychopathological profiles, as measured by the SCL-90R, were associated with the group characterized by the highest compensatory exercise levels, while the most favorable psychopathological profiles were displayed by patients in the group characterized by mild–moderate compensatory exercise levels. With regard to personality profiles, the groups exhibited differences in the harm avoidance (highest mean for patients with high compensatory exercise levels) and persistence scale (highest mean for patients with low compensatory exercise levels).

### 3.3. Comparisons Regarding Therapy Outcomes between the Groups

Table 3 contains the treatment outcomes in each group and the pairwise comparisons (contrasts). The upper part compares the risk of dropout, non-remission, partial remission, or total remission between the groups, and the lower part compares the risk of good versus poor treatment outcomes (poor responses were considered for dropout or non-remission). The highest risk for a poor therapy outcomes was obtained for individuals with high levels of compensatory exercise (63.7%), followed by those with mild to moderate levels (58.3%). Conversely, the group with the lowest levels of compensatory exercise was associated with the highest rates of favorable outcomes (see Table 3).

The logistic model which contains the predictors of the therapy outcomes is displayed in Table 4. This model retained and modeled the variables that achieved statistical significance for the prediction of poor therapy outcomes (dropout or non-remission). Lower values in the BMI indexes, TCI-R reward dependence, and TCI-R self-directedness increased the likelihood of a poor result, in a manner similar to higher levels of compensatory exercise.

### 3.4. Path Analysis

**Figure 1:**
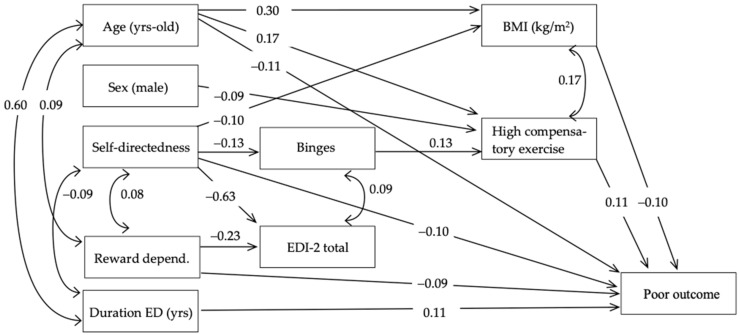
Path analysis: standardized coefficients in the SEM. Note. BMI: body mass index, ED: eating disorder, EDI-2: Eating Disorders Inventory-2. Only significant coefficients are retained in the model.

Figure 1 contains the path diagram for the SEM, with the standardized coefficients measuring the relationships between the variables. Adequate goodness of fit was achieved (RMSEA = 0.053 [95% confidence interval: 0.032 to 0.073]; CFI = 0.943; TLI = 0.901; and SRMR = 0.033), and the global predictive capacity was around 54% (CD = 0.544).

The increased risk of poor outcomes was directly related with younger age, longer duration of the ED-related problems, and lower scores in the self-directedness and reward dependence scales. The high compensatory exercise levels achieved a mediational role in different paths. Firstly, being a woman and of older age were both related with higher exercise levels, which subsequently increased the risk of a poor outcome. Secondly, lower self-directedness increased the number of binges, and this path next contributed to increased compensatory exercise levels, which then contributed to a higher likelihood of a poor outcome. The BMI was also a mediational variable in the subsequent path: older age and lower self-directedness were related to increased BMI, which next predicted a lower risk of a poor outcome.

## 4. Discussion

The present study aimed to investigate the effect of high levels of compensatory exercise on clinical variables and therapy outcomes in BN and OSFED-BN. Overall, our findings indicate that those patients who engaged in high levels of compensatory exercise, next to exhibiting higher eating-related psychopathology, general psychopathology, and more dysfunctional personality traits also had an increased likelihood of a poor outcome.

Both BN and OSFED-BN have a multifactorial etiology, which includes specific psychological factors and personality traits [13]. The sample analyzed in this study exhibited similar characteristics as those observed in previous research, such as impulsivity and affective instability [40], high neuroticism, harm avoidance, and novelty seeking [41,42]. When we focused on those with the highest levels of compensatory exercise levels, we observed that they exhibited greater levels of harm avoidance (characterized by anxiety, insecurity, worry, and fear), whereas the lowest levels of compensatory exercise were associated with higher levels of persistence (high levels of perseverance and rigidity). Harm avoidance appears strongly associated with both depression and anxiety [43,44] and has been thought to serve as a gateway to ED [45,46]; and specifically in patients with BN, weight management efforts may promote excessive exercise [47]. Low levels of persistence could also be associated with poor treatment outcomes due to difficulties maintaining a focus on set treatment goals [48].

As we hypothesized a priori, and in accordance with the findings of other studies that analyzed maladaptive exercise behaviors [24,49,50,51], the patient group exhibiting high levels of compensatory exercise demonstrated elevated levels of total eating disorder symptomatology. We observed elevated levels of bulimic symptoms as well as higher impulsivity, social insecurity, feelings of emptiness and insecurity, and difficulties in recognizing emotional states. No significant differences were observed between the three groups in terms of body dissatisfaction and drive for thinness. It is noteworthy that the patients within the highest compensatory exercise group also reported a higher frequency of binge episodes per week, which may have been a contributing factor to the elevated mean BMI observed in this group.

Our results also identified the most unfavorable psychopathological profiles to be associated with the group characterized by the highest compensatory exercise levels. These patients exhibited greater anxiety, depression, obsessive symptoms, and overall psychological emotional distress. Negative affectivity may act as a trigger and maintenance factor for compensatory exercise [52] rather than as a motivator for weight control. This may differ from individuals with restrictive anorexia nervosa, in whom the need for weight control and body shape are more predominant [50].

Indeed, maladaptive personality traits, psychiatric comorbidities, and psychological distress, frequently observed in individuals with ED, contribute to longer courses of the disorders and poor treatment outcomes [53,54,55]. Our results appear to be consistent with the assumption that compensatory exercise represents a core symptom of both BN and OSFED-BN [20] and is positively associated with clinical severity [23,49,51]. In this context, our findings indicated that individuals with high levels of compensatory exercise exhibited the highest risk for a poor therapy outcome, while those with the lowest levels of compensatory exercise displayed the highest likelihood of favorable outcomes. Moreover, higher levels of compensatory exercise proved to be a predictor of poor treatment outcomes.

Individuals who engage in compensatory exercise place emphasis on regulating their body shape and controlling their dietary habits, leading to a vicious cycle of reinforcing weight control behaviors [56]. Over time, individuals may rely on compensatory exercise as a means of regulating their emotions, resulting in a dependency on these behaviors and the establishment of a self-sustaining ED [52]. Previous studies have indicated that excessive exercise is associated with prolonged hospitalizations and increased suicidal behavior in patients with ED [22,23]. Some studies in the literature—the majority of which, however, have focused on anorexia nervosa—have demonstrated that a high load of physical activity and maladaptive exercise is associated with the presence of psychological and medical comorbidities, which in turn negatively impact the outcome of ED [57]. Further evidence is needed to establish this association in the other types of ED.

Interestingly, the path analysis showed that lower self-directedness increased the likelihood of binges, which contributed to increased compensatory exercise levels rather than contributing to a higher likelihood of a poor treatment response. Those with lower levels of self-direction are often perceived as irresponsible, unreliable, and unable to define, set, and pursue meaningful internal goals. They lack an internal organizing principle, which can lead to difficulties in navigating social interactions and maintaining consistent behaviors and achievement of own goals [45]. This personality trait may influence the number of binge episodes, which in turn may lead to an attempt to cope by increasing the hours of compensatory exercise. Further research is needed to consider how to shape psychotherapeutic approaches to adequately address these aspects during ED treatment.

The results of this study may contribute to the development of targeted interventions for patients with bulimic-related disorders. Physical activity and exercise may improve the symptoms of mental disorders, including associated comorbidities and cognitive deficits [58], a finding that has been corroborated for ED as well [59,60,61]. Interestingly, patients showing mild–moderate levels of compensatory exercise had more favorable general psychopathological profiles throughout different subscales. This may imply that moderate exercise could have a beneficial effect, despite its compensatory nature. Incorporating adapted and supervised exercise programs into the treatment of ED may help to improve symptoms such as anxiety, depression, or medical complications (e.g., dyslipidemia, diabetes, and cardiovascular disease). Further research is needed in this regard. Nonetheless, it is important to consider the potential limitations of this approach, such as in patients with a low BMI trying to gain weight. To alleviate a high urge for physical activity when activity is medically not possible, newer interventions using VR technology have been suggested as possible solutions [62].

This study is subject to certain limitations. Firstly, the self-reported nature of the variable assessing compensatory exercise levels introduces a potential source of bias. The lack of precise information (i.e., obtained from accelerometry methods) represents a limitation in the exact quantification of the number of hours of compensatory exercise. However, the information collected in this study was gathered during an in-depth interview conducted by experienced clinical psychologists specialized in ED with the objective of ascertaining data as close to reality as possible. Secondly, our analysis lacks an examination of different subtypes of activity, including adaptive exercise, and instead focuses only on compensatory exercise. In recent years, research has sought to categorize dimensions of maladaptive exercise in terms of “excessive exercise”, “compulsive exercise”, and “compensatory exercise” [20,24,63]. Different motivations may promote the different forms of exercise, e.g., the desire to burn calories, the goal of emotional regulation, the participation in social activities, or even the mere sake of enjoyment. Further investigation is necessary in order to comprehend the effects of each exercise type and the motivation behind it on treatment and outcomes. Finally, the generalizability of our results may be limited; for instance, the sample only included patients with BN and OSFED-BN, which, although they represent a core population in the engagement of compensatory exercise, limits the extrapolation of our results to other ED types. Finally, the low representation of men in the sample did not permit a sex comparisons, thus limiting the ability to draw conclusions regarding this perspective.

## 5. Conclusions

The present study confirms an association between high levels of compensatory exercise and a more severe course of BN and OSFED-BN, including higher eating disorder psychopathology, general psychopathology, and more dysfunctional personality traits. In turn, high levels of compensatory exercise increased the likelihood of a poor treatment outcome. It is noteworthy that patients exhibiting mild-to-moderate levels of compensatory exercise exhibited lower psychological distress. This may indicate that moderate exercise could exert a beneficial effect, despite its compensatory nature. In light of these findings and considering the potential benefits of physical activity in the context of mental health, there is a need to develop interventions aimed at increasing adaptive exercise patterns in people with ED, such as bulimia spectrum disorders. Further research is required to determine how psychotherapeutic approaches can be optimized to adequately address these aspects during the treatment of ED and to develop guidelines for the assessment and management of exercise in populations with ED. The involvement of an exercise professional with specialized training in ED as part of an interdisciplinary treatment team may provide an efficient approach to addressing these issues.

## Figures and Tables

**Table 1:** Descriptive for the variables in this study.

	Total N = 478	
	n	%
Sex		
Women	440	92.1%
Men	38	7.9%
	Mean	SD
Age (years)	29.51	9.57
Age of onset ED (years)	19.54	7.85
Duration of the ED (years)	10.10	8.23
BMI (kg/m^2^)	25.50	6.48
Binge episodes (number/week)	5.58	5.60
Purge episodes (number/week)	5.37	7.18
SCL-90R: global severity index (GSI)	1.85	0.71
SCL-90R: positive symptom total (PST)	66.82	15.73
SCL-90R: positive symptom distress index (PSDI)	2.41	0.56
EDI-2: Total score	112.57	41.88
TCI-R: Novelty seeking	104.15	16.43
TCI-R: Harm avoidance	118.41	20.42
TCI-R: Reward dependence	101.18	15.31
TCI-R: Persistence	108.33	20.99
TCI-R: Self-directedness	111.71	19.89
TCI-R: Cooperativeness	132.33	15.53
TCI-R: Self-transcendence	65.29	14.69
	n	%
Compensatory exercise level		
High	270	56.5%
Mild–moderate	84	17.6%
Low	124	25.9%
	n	%
Outcome		
Dropout	235	49.2%
Non-remission	51	10.7%
Partial remission	94	19.7%
Total remission	98	20.5%
Outcome		
Good	192	40.2%
Poor	286	59.8%

Note. BMI: body mass index, ED: eating disorder, EDI-2: Eating Disorders Inventory-2, SCL-90R: Symptom Checklist-90-Revised, SD: standard deviation, TCI-R: Temperament and Character Inventory-Revised. Good outcome: total remission or partial remission. Poor outcome: dropout or non-remission.

**Table 2:** Group comparisons based on compensatory exercise levels: clinical profiles at baseline.

	High *N* = 270		Mild– Moderate *N* = 84		Low *N* = 124		High vs. Mild– Moderate		High vs. Low		Mild– Moderate vs. Low	
	Mean	SD	Mean	SD	Mean	SD	*p*	*|d|*	*p*	*|d|*	*p*	*|d|*
Age (years)	31.03	9.87	27.79	9.10	27.37	8.67	0.006 *	0.34	0.001 *	0.39	0.756	0.05
Age of onset ED (years)	19.96	8.40	18.68	7.20	19.23	6.98	0.193	0.16	0.391	0.09	0.622	0.08
Duration of the ED (years)	11.25	8.47	9.08	7.34	8.26	7.89	0.032 *	0.28	0.001 *	0.37	0.480	0.11
BMI (kg/m^2^)	26.61	7.55	24.70	5.39	23.62	3.41	0.017 *	0.29	0.001 *	**0.51 †**	0.227	0.24
Binges (number/week)	6.36	6.61	4.26	3.09	4.80	4.04	0.003 *	0.41	0.010 *	0.28	0.493	0.15
Purges (number/week)	5.70	7.96	5.99	7.29	4.21	4.85	0.751	0.04	0.055	0.23	0.079	0.29
EDI-2: Drive for thinness	15.67	4.66	15.85	4.28	16.52	4.49	0.759	0.04	0.084	0.19	0.292	0.15
EDI-2: Body dissatisfaction	18.74	7.29	17.04	7.43	17.55	7.82	0.068	0.23	0.141	0.16	0.627	0.07
EDI-2: Interoceptive awareness	13.10	6.88	11.17	7.09	13.44	7.33	0.028 *	0.28	0.660	0.05	0.023 *	0.31
EDI-2: Bulimia	10.36	4.86	9.25	4.76	9.20	5.31	0.073	0.23	0.032 *	0.23	0.945	0.01
EDI-2: Interpersonal distrust	6.19	4.72	5.04	4.76	5.65	4.52	0.050 *	0.24	0.287	0.12	0.357	0.13
EDI-2: Ineffectiveness	12.69	7.56	9.83	6.41	11.34	7.47	0.002 *	0.41	0.091	0.18	0.148	0.22
EDI-2: Maturity fears	8.93	5.85	7.57	5.47	7.74	5.64	0.059	0.24	0.057	0.21	0.833	0.03
EDI-2: Perfectionism	5.98	4.43	5.87	4.82	6.68	4.55	0.843	0.02	0.158	0.15	0.208	0.17
EDI-2: Impulse regulation	7.73	6.27	6.04	5.34	7.82	6.35	0.028 *	0.29	0.885	0.02	0.040 *	0.30
EDI-2: Asceticism	8.20	3.82	6.79	3.73	8.27	4.31	0.004 *	0.37	0.862	0.02	0.008 *	0.37
EDI-2: Social insecurity	8.63	5.27	7.35	5.33	7.77	5.00	0.049 *	0.24	0.127	0.17	0.568	0.08
EDI-2: Total score	116.21	41.76	101.77	39.00	111.98	43.03	0.006 *	0.36	0.349	0.10	0.084	0.25
SCL-90R: Somatization	1.99	0.91	1.69	0.88	1.73	0.86	0.007 *	0.34	0.008 *	0.29	0.728	0.05
SCL-90R: Obsessive	2.12	0.80	1.81	0.75	2.03	0.84	0.003 *	0.39	0.308	0.11	0.058	0.27
SCL-90R: Sensitivity	2.23	0.90	1.86	0.86	2.18	0.86	0.001 *	0.41	0.617	0.05	0.012 *	0.37
SCL-90R: Depressive	2.43	0.87	2.05	0.89	2.30	0.86	0.001 *	0.43	0.176	0.15	0.043 *	0.28
SCL-90R: Anxiety	1.87	0.88	1.59	0.90	1.76	0.86	0.010 *	0.32	0.228	0.13	0.178	0.19
SCL-90R: Hostility	1.57	1.03	1.19	0.87	1.41	0.91	0.002 *	0.39	0.136	0.16	0.113	0.24
SCL-90R: Phobic anxiety	1.22	0.94	1.00	0.85	1.10	0.90	0.059	0.24	0.225	0.13	0.457	0.11
SCL-90R: Paranoia	1.55	0.87	1.36	0.81	1.55	0.80	0.074	0.22	0.978	0.00	0.109	0.24
SCL-90R: Psychotic	1.47	0.77	1.25	0.66	1.46	0.78	0.022 *	0.30	0.926	0.01	0.050 *	0.29
SCL-90R: global severity index (GSI)	1.93	0.71	1.64	0.71	1.82	0.69	0.001 *	0.42	0.156	0.16	0.059	0.27
SCL-90R: positive symptom total (PST)	68.21	15.23	63.29	18.22	66.18	14.67	0.012 *	0.29	0.232	0.14	0.192	0.17
SCL-90R: positive symptom distress index (PSDI)	2.47	0.55	2.21	0.54	2.41	0.55	0.001 *	0.47	0.332	0.10	0.011 *	0.36
TCI-R: Novelty seeking	103.46	17.25	104.48	14.86	105.42	15.64	0.621	0.06	0.273	0.12	0.685	0.06
TCI-R: Harm avoidance	121.68	20.29	116.74	20.41	112.44	19.36	0.049 *	0.24	0.001 *	0.47	0.130	0.22
TCI-R: Reward dependence	101.55	14.74	101.58	16.58	100.11	15.72	0.985	0.00	0.389	0.09	0.498	0.09
TCI-R: Persistence	106.00	21.98	106.73	19.49	114.49	18.57	0.780	0.03	0.001 *	0.42	0.008 *	0.41
TCI-R: Self-directedness	110.34	20.84	112.29	19.06	114.31	18.10	0.433	0.10	0.065	0.20	0.470	0.11
TCI-R: Cooperativeness	132.09	16.18	132.21	13.43	132.91	15.51	0.950	0.01	0.628	0.05	0.751	0.05
TCI-R: Self-transcendence	65.05	15.30	65.11	12.50	65.94	14.82	0.976	0.00	0.577	0.06	0.688	0.06

Note. BMI: body mass index, ED: eating disorder, EDI-2: Eating Disorders Inventory-2, SCL-90R: Symptom Checklist-90-Revised, SD: standard deviation, TCI-R: Temperament and Character Inventory-Revised. * Bold: statistically significant comparison. † Effect size within the range mild–moderate to high–large.

**Table 3:** Group comparisons based on activity levels: treatment outcomes.

	High *N =* 270		Mild– Moderate *N =* 84		Low *N =* 124		High vs. Mild– Moderate		High vs. Low		Mild– Moderate vs. Low	
	*n*	*%*	*n*	*%*	*n*	*%*	*p*	*|C*-*V|*	*p*	*|C*-*V|*	*p*	*|C*-*V|*
Outcome												
Dropout	144	53.3%	41	48.8%	50	40.3%	0.824	0.051	0.115	0.123	0.629	0.091
Non-remission	28	10.4%	8	9.5%	15	12.1%						
Partial remission	48	17.8%	16	19.0%	30	24.2%						
Total remission	50	18.5%	19	22.6%	29	23.4%						
Outcome												
Good	98	36.3%	35	41.7%	59	47.6%	0.375	0.047	0.034 *	0.107	0.400	0.058
Poor	172	63.7%	49	58.3%	65	52.4%						

Note. * Bold: statistically significant comparison. Good outcome: remission or partial remission. Poor outcome: dropout or non-remission.

**Table 4:** Stepwise logistic regression with predictors of poor therapy outcomes.

Independent Variable (Predictor)	B	SE	*p*	OR	95%CI OR		NR^2^	H-L
BMI (kg/m^2^)	−0.038	0.015	0.011	0.962	0.934	0.991	0.060	0.147
High compensatory exercise	0.284	0.114	0.013	1.328	1.063	1.659		
TCI-R reward dependence	−0.013	0.006	0.047	0.988	0.975	1.000		
TCI-R self-directedness	−0.013	0.005	0.011	0.988	0.978	0.997		

Note. BMI: body mass index, OR: odds ratio, SE: standard error, TCI-R: Temperament and Character Inventory-Revised. 95%CI: 95% confidence interval. NR^2^: Nagelkerke’s pseudo R2 coefficient. H-L: Hosmer and Lemeshow test (*p*-value).

## Data Availability

All inquiries regarding availability of the data should be referred to the corresponding author (F.F.-A.), as there are ongoing studies using the data and to preserve patient confidentiality. Requests will be considered on a case-by-case basis.
